# Lightweight Glass Fiber-Reinforced Polymer Composite for Automotive Bumper Applications: A Review

**DOI:** 10.3390/polym15010193

**Published:** 2022-12-30

**Authors:** Hossein Mohammadi, Zaini Ahmad, Saiful Amri Mazlan, Mohd Aidy Faizal Johari, Geralt Siebert, Michal Petrů, Seyed Saeid Rahimian Koloor

**Affiliations:** 1 Faculty of Mechanical Engineering, Universiti Teknologi Malaysia, Johor Bahru 81310, Malaysia; 2 Engineering Materials and Structures (eMast) Ikohza, Malaysia–Japan International Institute of Technology (MJIIT), Universiti Teknologi Malaysia, Kuala Lumpur 54100, Malaysia; 3 Department of Civil Engineering and Environmental Sciences, Institute for Structural Engineering, Universität der Bundeswehr München, Werner-Heisenberg-Weg 39, Neubiberg, 85579 Munich, Germany; 4 Faculty of Mechanical Engineering, Technical University of Liberec (TUL), Studentská 1402/2, 461 17 Liberec, Czech Republic

**Keywords:** automotive bumper beam, polymer matrix, glass fiber, mechanical design, impact energy: energy absorption

## Abstract

The enhancement of fuel economy and the emission of greenhouse gases are the key growing challenges around the globe that drive automobile manufacturers to produce lightweight vehicles. Additionally, the reduction in the weight of the vehicle could contribute to its recyclability and performance (for example crashworthiness and impact resistance). One of the strategies is to develop high-performance lightweight materials by the replacement of conventional materials such as steel and cast iron with lightweight materials. The lightweight composite which is commonly referred to as fiber-reinforced plastics (FRP) composite is one of the lightweight materials to achieve fuel efficiency and the reduction of CO_2_ emission. However, the damage of FRP composite under impact loading is one of the critical factors which affects its structural application. The bumper beam plays a key role in bearing sudden impact during a collision. Polymer composite materials have been abundantly used in a variety of applications such as transportation industries. The main thrust of the present paper deals with the use of high-strength glass fibers as the reinforcing member in the polymer composite to develop a car bumper beam. The mechanical performance and manufacturing techniques are discussed. Based on the literature studies, glass fiber-reinforced composite (GRP) provides more promise in the automotive industry compared to conventional materials such as car bumper beams.

## 1. Introduction

The automotive bumper beam is the back or front part of the vehicle which is used as protection for the passengers inside the vehicle during an impact collision. In addition, it plays a vital role in the energy-absorbing capacity of the vehicle during the collision. The bumpers are focused on mitigating the injury to the pedestrians struck by the vehicles but they are incapable of decreasing the impact effects at high speed [1].

The type of material plays a key role in efficient energy adsorption during the collision. Metallic energy adsorbers have been widely used in automotive applications for more than two decades [2,3]. Thus, a key concern regarding automotive bumpers is the selection of material. In this regard, better performance compared to previous materials, cost-effectiveness, enhanced strength, and weight reduction should be taken into consideration when selecting bumper beam materials [4].

The substitution of materials is one of the approaches to reduce the weight of the vehicle in which the steel alloys in automotive applications are substituted with lighter weight-saving materials such as polymer composite. In the automotive industry, the bumper beam has the potential to remarkably reduce weight by using lightweight materials such as composite materials. In general, they are comprised of two components which are the matrix and the reinforcement. The high-strength fibers are embedded in a matrix with a distinct interface between them and act as load-carrying constituents while the matrix is the principal load transfer medium which keeps them in the desired orientation. Composite materials have found abundant applications in civil and transportation industries [5] which are mainly related to lightweight, suitable mechanical performance, resistance against harsh environmental conditions as well as practical low-cost production. Most bumper beams have previously been made up of steel. Nonetheless, conventional materials such as aluminum and metals were replaced with polymer composites [6,7,8]. They offer advantages over their metallic counterparts such as lower density, higher strength, higher impact energy-absorbing capacity, and ease of producing complex shapes.

The weight reduction (lightweight) of automotive structures can be attained by utilizing composite materials ranging from carbon-reinforced plastic (CFRP) composite to glass fiber-reinforced plastics (GFRP) composite [9,10]. Such materials provide less weight than metals including iron, steel, and aluminum which dominate the automotive industry. It was reported that replacing the metallic materials with GFRP composite causes 40–60% weight saving while retaining the stiffness and strength [11]. Additionally, the lightweight increases fuel efficiency through low discharge of carbon dioxide (CO_2_) from engine emissions [12] which is economically important to industries in compliance with global legislation [13]. For instance, a mass reduction of 100 kg resulted in a saving of 0.15–0.7 fuel per 100 km [14]. Besides, weight reduction is beneficial for energy-absorbing capability and impact resistance [15]. Thus, many attempts have been made to employ fiber-reinforced plastic (FRP) composite in the interior parts of automobile parts that sustain heavy loads during different driving circumstances [16,17]. Nonetheless, the effective cost and the high mechanical properties need to be addressed for their general acceptance and efficient application as the major drawback in automobile and structural application is the low impact resistance [18]. Therefore, the objective of this review paper is to investigate the impact resistance of GFRP composite as part of a car bumper assembly.

## 2. Role of Composite in the Automotive Industry

As the world is seeking ways to enhance fuel efficiency and ensure the safety of the automobile, the weight reduction of the material is crucial. In addition, the mass reduction of automobiles reduces CO_2_ emissions [19]. The European Union Commission has established the European Guidelines 2000/53/RG for the automobile industries for machine recyclability. Countries such as Japan and the USA are emphasizing the recyclability of automobiles [20].

**Figure 1:**
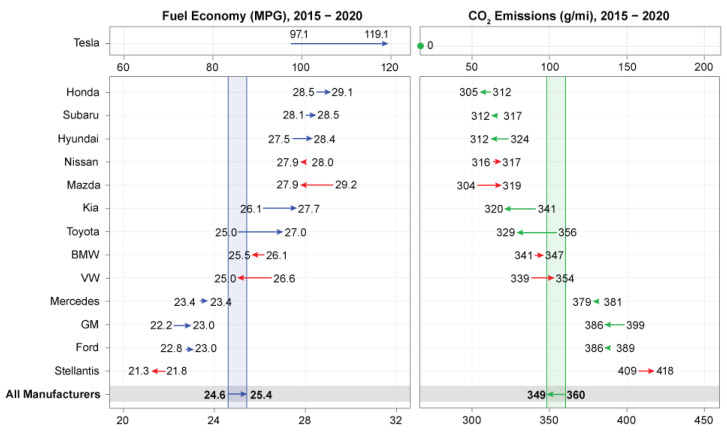
Performance of automobile manufacturers in reducing CO_2_ emission and improving fuel consumption from 2013–2015 [21].

Automobile fuel consumption and the emission of CO_2_ are the main challenges that automobile manufacturers are facing nowadays. This is because engine emission is known as the main base of air pollution [12]. One way to advance fuel efficiency and reduce CO_2_ emissions is weight reduction. This is because the technology for improving fuel efficiency is restricted. It was previously reported that the elimination of every 10% of the vehicle’s total weight causes a 7% improvement in fuel economy [14]. Besides, weight reduction is beneficial for energy-absorbing capability and impact resistance [15]. Automobile manufacturers have positively reacted to air pollution which is shown in Figure 1. It is observed that the CO_2_ emission and the fuel economy decreased and increased over five years, respectively [21]. In Figure 1, MPG stands for Miles Per Gallon. Figure 1 shows the fuel economy (MPG) and CO_2_ emission in terms of grams per mile (g/mile) for car manufacturers between the years 2015–2020. For example, the largest reduction in CO_2_ emission was ascribed to Toyota at 27 g/mi which is shown by the green arrow, and the fuel economy was increased from 25 MPG to 27 MPG which is shown by the blue arrow. In contrast, Volkswagen revealed the largest increase in CO_2_ emission by 15 g/mi and showed a decrease in fuel economy which are shown by red arrows. There are different means to reduce the weight of automobile parts including structural optimization [22], enhancement of the functionality of the component [23], and the employment of lightweight material [11]. Among these approaches, employing lightweight material is the most effective. In this regard, many attempts have been made to modify automobile constituents by replacing the metal parts with lightweight materials such as aluminum (Al) or alloy, or fibrous polymer composites [16]. Zhang et al. [24] have provided a review of lightweight materials for automobile applications.

The composite materials in which two or more desired materials are combined to overwhelm the disadvantage of each alone have found wide applications in automotive sectors. This is ascribed to their high specific strength, design flexibility, corrosion resistance, as well as thermal conduction. They can also contribute to a weight reduction of 15% to 40% in automobiles [24]. Thus, GFRP composite is of interest to automotive manufacturers due to its low weight and high performance [25,26,27]. In automobile applications, the material should withstand extreme environments such as sudden impact and variable loads. Thus, the material should possess high strength, low density, damage tolerance [28], as well as processability. Such exceptional properties cannot always be found in solitary materials.

Government regulations covering lightweight, fuel efficiency, CO_2_ emissions as well as crash performance should be considered in the selection of composite materials. Finally, cost and quality characteristics as customer requirements are also important [29]. In an automobile, a 10% overall weight reduction causes an increase of approximately 7% in fuel efficiency [30]. The inertia forces can also be overcome by overall weight reduction which consequently saves on the power required for acceleration and braking. Further, weight reduction can be beneficial for the automobile and its stability. Nonetheless, the only obstacle in replacing conventional materials with superior lightweight materials is the high cost.

The resistance to deformation under impact collision offering safety to passengers in the automobile is referred to as crashworthiness. It is a measure of material for the absorption of impact energy while maintaining the structure against plastic deformation. According to reports provided by the World Health Organization (WHO), approximately 1.35 million people die in road car accidents annually [30]. Automobile manufacturers nowadays focus on the safety and eco-friendliness of vehicles [31]. The matrix material in a composite takes the compressive loads while the reinforcing phase carries the tensile load. This causes resistance to sudden impact during a collision, retaining the high elastic strength of the material. Some examples of composite materials (particularly GFRP composite) are shown in Table 1.

## 3. Polymer Composite Materials

Composite materials are defined as artificial multiphase materials which are comprised of continuous and dispersed phases. The composite materials show superior mechanical properties due to the incorporation of desirable properties of each constituent [38,39]. The factors that determine the final properties of the composite are the composition of the matrix and the alignment of reinforcements [40]. The mechanical efficiency of the composite materials is known to be a function of the reinforcing constituent. Nonetheless, the matrix phase also significantly contributes to the mechanical performance [41]. The continuous material phase is also called the matrix and the dispersed phase is called reinforcement [42]. The matrix in composite materials usually has a ductile behavior and could be a polymer, ceramic, and metallic. The reinforcing phase can provide strength to the composite through load transfer. This is because the fragmented ends of fibers are pulled apart and the shear forces are exerted on the matrix phase which leads to a slow and gradual development of stress on the fragments. This enables the composite to endure more stress without fracture. In short, the strength and toughness of the composite are enhanced by the synergetic effects of the matrix and fibers [43]. The distribution of stress from matrix to fibers has a direct influence on the mechanical properties of composite material [44]. In this regard, a suitable stress transfer in composite material is guaranteed through the fractions of fibers and inter-ply adhesion across the matrix as well as the reinforcing phases [45].

The composite can be classified according to its reinforcement type such as particle-reinforcement, fiber-reinforcement, etc. [46]. Therefore, the enhancement of key properties in polymer composite materials such as strength, stiffness, and lower cost is necessary owing to their various applications. Natural and synthetic fibers are the two types of fibers used as reinforcement in the manufacturing of composite materials. Synthetic fibers, also known as manufactured fibers, are commonly used to make fiber-reinforced polymer composites in engineering industries. In this regard, 90% of the global market has been taken by glass fibers to fabricate composite materials in the industry [47]. Synthetic fibers can be used in various forms in the automobile industry including multilayer structures, woven and non-woven structures, wrap, as well as circular knitted, respectively. In addition, the impact resistance of synthetic fiber-reinforced composite is higher than that of natural fiber-reinforced composite counterparts [48].

The presence of fibrous reinforcement provides strength and rigidity bearing structural strain [49]. Different advantages of synthetic fibers such as glass are high strength and impact resistance. Nonetheless, they possess poor biodegradability, and recyclability [50]. On the other hand, the benefits of natural fibers over synthetic ones are highlighted as low cost and density, biodegradability, and eco-friendly [51,52]. However, the variability of their characteristics is their key weakness. The absorption of moisture and poor mechanical performance are among the disadvantages of natural fiber-reinforced polymer composite [53,54,55]. The mechanical properties of natural fibers are deteriorated by the presence of impurities including hemicellulose, lignin, and pectin on their surface, as the interfacial adhesion bonding at the microscale between the matrix phase and reinforcing constituents is hampered. Additionally, the hydrophilicity of the natural fibers and the hydrophobicity of the polymer matrix causes poor interfacial adhesion bonding. Thus, chemical treatment is required for the natural fibers to reduce their hydrophilicity and the presence of impurities. Reddy et al. [56] reviewed the effect of chemical treatment on natural fiber in designing fiber-reinforced composite. An assessment between the natural fiber and synthetic fiber is tabulated in Table 2.

**Figure 2:**
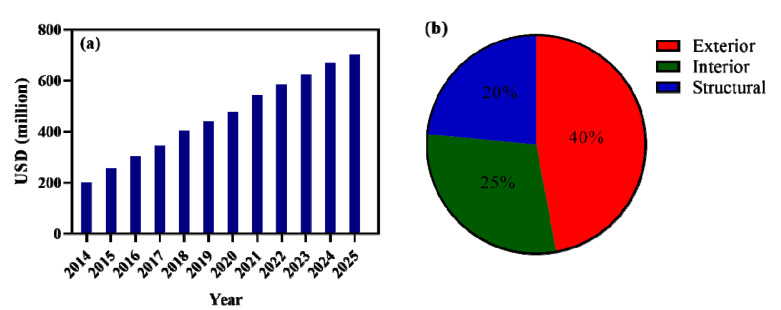
(**a**) The market revenue for polymer matrix composite in the United States from 2014–2025. Reprinted with permission [59]. (**b**) The pattern of a global application of polymer matrix composite in different automotive constituents. Reprinted with permission [24].

The use of polymer matrix composite in automotive market revenue has continuously grown in the last decades as shown in Figure 2a. It was also revealed that most of the polymer matrix composite, approximately 65%, was used in the exterior and interior parts of automobiles, as shown in Figure 2b. Based on the report by market, the automotive polymer composite industry in 2014 was found to be valued at USD 200 million and it will probably reach USD 700 million by 2025 [59].

### Petroleum-Based Polymer Matrix

The petroleum-based polymer resin is classified as a chemical product that is found in fossil fuels such as oil or coal [48]. Thermoplastics and thermosets are types of petroleum-based matrices used for the fabrication of composite materials. Thus, the matrix materials in the polymer composite can be classified as thermosetting and thermoplastic. The selection of a suitable matrix based on the intended application is a critical issue as the ultimate properties of the composite are directly affected by the matrix material [60]. The thermoplastic polymers comprising nylon [61], polystyrene [62], polypropylene [63], polyethylene [64], polyether ether ketone [65], and polyvinylchloride [66] have high molecular weight and a low melting point, thus can be reformed and melted several times upon the application of heat (physical change) without chemical reaction. Additionally, they are solid at room temperature and have a high viscosity which makes their processing difficult. Therefore, they are manufactured in filament forms. Thermoplastics are also cheap, reusable, and suitable for mass production in the automobile industry and exhibit a longer storage life when compared to thermosetting polymers [27]. The impact resistance, damage tolerance, and reformability of thermoplastics are higher than that of thermoset resin [67,68].

On the other hand, thermosetting polymers comprising polyester resin [69], polyurethane [70], epoxy resin [71], phenolic [72], and vinyl ester resin [73] possess low molecular weight and cannot be reformed by heating. The three-dimensional covalent bonding connects the polymer chains in thermosetting polymers. They are also considered insoluble and infusible materials that have been cured through heat or catalyst processes [74]. This type of matrix shows brittleness and low fracture toughness at room temperature. Epoxy resin has been used in the automobile sectors due to its mechanical strength, excellent impact, low shrinkage as well as surface texture. Nonetheless, the implementation of epoxy for mass production in the automobile industry is restricted due to prolonged curing cycles. In contrast, vinyl ester resin shows a better energy-absorbing capacity with low curing time. Therefore, glass fiber-reinforced vinyl ester resin has been employed in a variety of large volume automobile applications [75]. A comparison between the physical and mechanical properties of thermosetting and thermoplastic matrices is tabulated in Table 3 and Table 4.

## 4. Glass Fiber-Reinforced Polymer Composite

The GFRP composite has been used in various forms in the automobile industry including continuous strands, long longitudinal glass fiber, woven fabric, non-woven mats, chopped strand mats, and veil mats [81]. This is attributed to their high specific stiffness and high specific strength in comparison to conventional metals [12,81]. The GFRP composite has been successfully used in various engineering applications [82]. The fibers, such as glass, possess a low modulus and lower cost while other fibers, such as carbon, have higher modulus and cost [83]. In this regard, the inexpensive fibers with low modulus not only yield a more tolerant hybrid composite against impact damage but also reduce the overall cost. In other words, the combination of high-stiffness fibers such as glass with materials with high strain-to-failure materials such as polyester, and polyamide can enhance the impact resistance of composites. This is attributed to the combination of impact resistance of ductile fiber with stiff reinforcement. The beneficial characteristics of GFRP composites are comprised of light weight, high strength, low cost, low density, and design flexibility [84]. The strength and modulus of fiber, volume content of fiber, and fiber/matrix interface bonding affect the mechanical properties of GFRP composite. The proper orientations and composition of fiber can provide functional properties to GFRP composites such as higher specific stiffness than aluminum and equal properties to steel [81].

### 4.1. Classification of GFRP Composites

In GFRP composites, the matrix is comprised of polyester, vinyl ester, phenolic and epoxy resin. They have attracted attention for their energy-absorbing applications [85,86]. The alkali glass (soda-lime glass) is a commonly available glass fiber that is abbreviated as A-glass. The main constituent to form the A-glass includes soda (Na_2_CO_3_), lime silica (Si_2_O_3_), alumina (Al_2_O_3_), sodium chloride (NaCl), and sodium sulfate. The compound glass is formed by the large amount of calcium borosilicate which is abbreviated as C-glass. The low dielectric constant glass which is formed by the existence of boron trioxide is abbreviated as D-glass. Electrical glass which is abbreviated as E-glass has been widely used in automobile applications due to its light weight, better strength, as well as higher stiffness. The constituent in the E-glass fiber is silica (SiO_2_), calcium oxide (CaO), magnesium oxide (MgO), sodium oxide (Na_2_O), potassium oxide (K_2_O), and boron trioxide (B_2_O_3_). There are different types of GFRP composites on the market including A-GFRP, C-GFRP, D-GFRP, E-GFRP, R-GFRP, and S-GFRP [84]. Nonetheless, 90% of the GFRP composites are represented by E-glass due to properties such as elastic modulus, flexibility, and strength [87]. Thus, the most commonly used GFRP composites to manufacture the structures for energy absorption are E-GFRP and S-GFRP [71,88,89]. These forms of glass fibers have been used as reinforcement in the composite in polyester, phenolic resins, and epoxy. The GFRP composite structures could be fabricated through various fabrication methods including mixing and molding, compression molding, and hand lay-up followed by hydraulic press and compression molding [84]. A summary of the mechanical and physical properties of different glass fibers is listed in Table 5.

### 4.2. Manufacturing Methods

Apart from the matrix and the reinforcing phase, manufacturing techniques can significantly affect the performance of composite materials [58]. Different systems of materials such as polymer resin, fiber, and particles, are involved in the manufacturing of composites which entails distinct processing tools and conditions. Additionally, the choice of manufacturing technique is reliant on various parameters including the dimension of the final composite part, production volume, and cost. The polymer industries attempt to develop new fabrication techniques which are capable of manufacturing high-quality composite parts at low cost. There are different methods to fabricate GFRP composite structures and some of the most common include wet forming, hand lay-up, injection molding, and compression molding, as well as additive manufacturing [84,91,92]. Injection molding extrusion and compression molding are used to make thermoplastic composite while hand layup and resin transfer molding are used to make the thermosetting composite. However, sheet molding compound (SMC) and bulk molding compound (BMC) can be used to produce GFRP composite. The former needs longer fibers while the latter requires shorter elements. Therefore, SMC is frequently used for the fabrication of larger parts whereby higher mechanical strength is desired. In this section, a brief description of these common methods is provided.

#### 4.2.1. Hand Lay-Up Method

**Figure 3:**
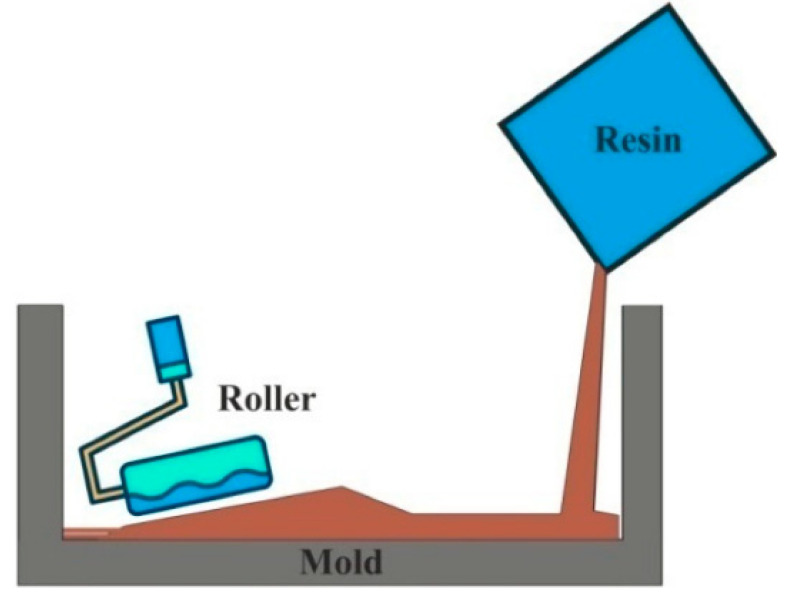
Schematic representation of hand lay-up technique. Reprinted with permission [38].

This method is the oldest open mold process in which the layers of fiber mat and resins are manually applied for the formation of laminated composite. The reinforcements can be found in the form of chopped strand mats or woven mats. The mold could also be found in the form of sheet metal, wood, or plastic. The advantage of this method is manufacturing composite parts of large size, and suitability for thermosets and thermoplastics. However, the cycling time is long, and the tooling cost and the volume of the production are low. A schematic of hand lay-up is shown in Figure 3.

#### 4.2.2. Injection Molding Method

**Figure 4:**
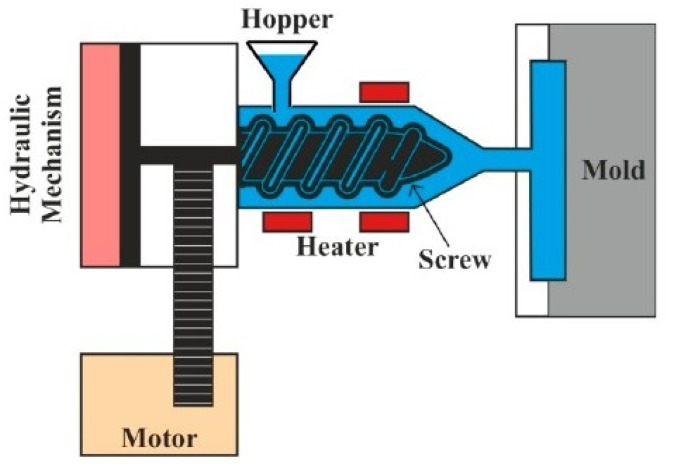
Schematic representation of injection molding technique. Reprinted with permission [38].

This technique is a closed mold process in which a mixture of polymer pellets or granules and short fibers are fed for splitting the mold cavity under high temperatures. The composite panel is finally removed from the mold through solidification using ejector pins. The advantage of this method is the high volume of production, production of complex shapes, and suitability for thermosets and thermoplastics. This technique is predominantly used for mass-producing composites. Nonetheless, the initial cost of this technique is high. The injection molding method is schematically depicted in Figure 4.

#### 4.2.3. Spray Lay-Up Method

**Figure 5:**
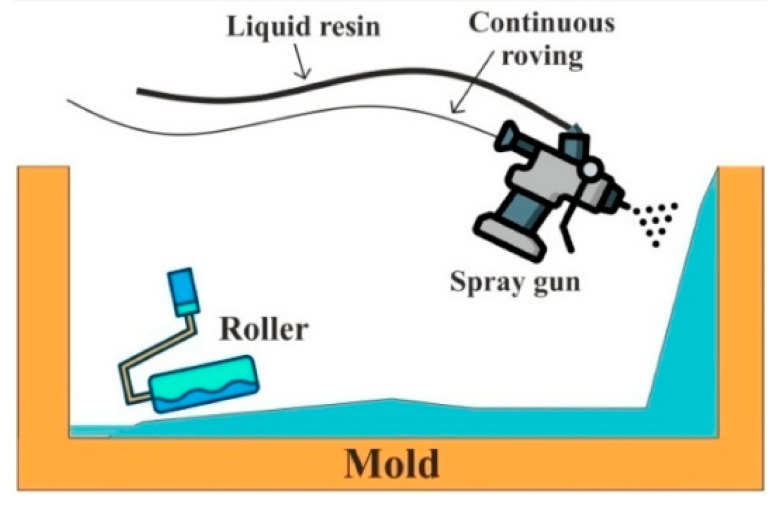
Schematic representation of spray lay-up. Reprinted with permission [38].

This technique is an open mold process in which a spray gun is used for spraying liquid resin (matrix phase) along with randomly oriented chopped fibers on the mold. The composite panel with complex geometry can be successfully prepared by this technique. The advantages of this method include the manufacturing of large parts and the suitability of thermosets and thermoplastics. Nonetheless, the mechanical properties of the product are low due to the application of chopped fibers. Additionally, the volume of the product and the tooling cost is low. The spray lay-up method is schematically depicted in Figure 5.

#### 4.2.4. Compression Molding Method

**Figure 6:**
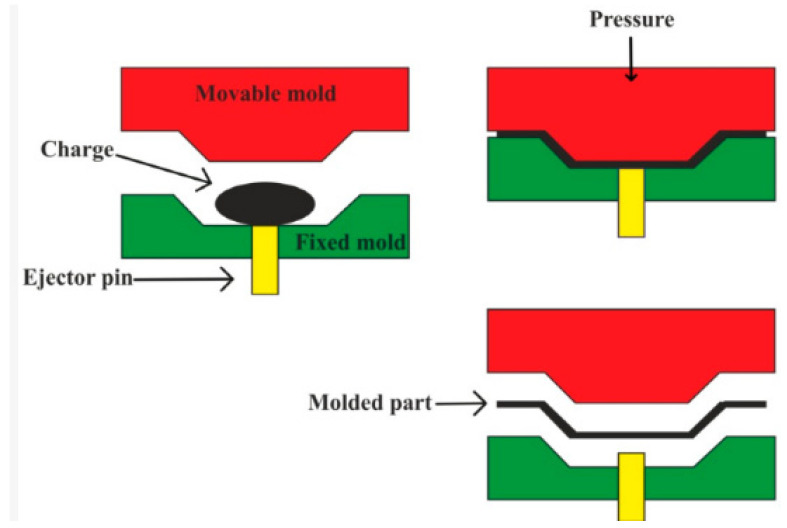
Schematic representation of compression molding. Reprinted with permission [38].

This is a closed mold process in which the pre-determined amount of material (a different constituent of the composite) is placed in between the upper and lower molds. The matrix and the reinforcement are mixed in a metallic mold with desired size and shape [93,94]. The mold is placed between the two heating platens under desired pressure and temperature according to the matrix material used throughout the fabrication process [95]. In the next stage, squeezing the under pressure and heat (for a time interval) leads to the formation of specified shapes. The composite is finally removed from the mold after curing at ambient temperature is carried out [67]. The advantage of this technique is in the high-volume production, short-time cycling, better control of the type and volume of fiber, and suitability for thermosets and thermoplastics. However, it is more expensive compared to the hand lay-up and spray lay-up techniques. It is used for the making of lightweight strong vehicle body panels. The compression molding method is schematically depicted in Figure 6.

#### 4.2.5. Resin Transfer Molding Technique

**Figure 7:**
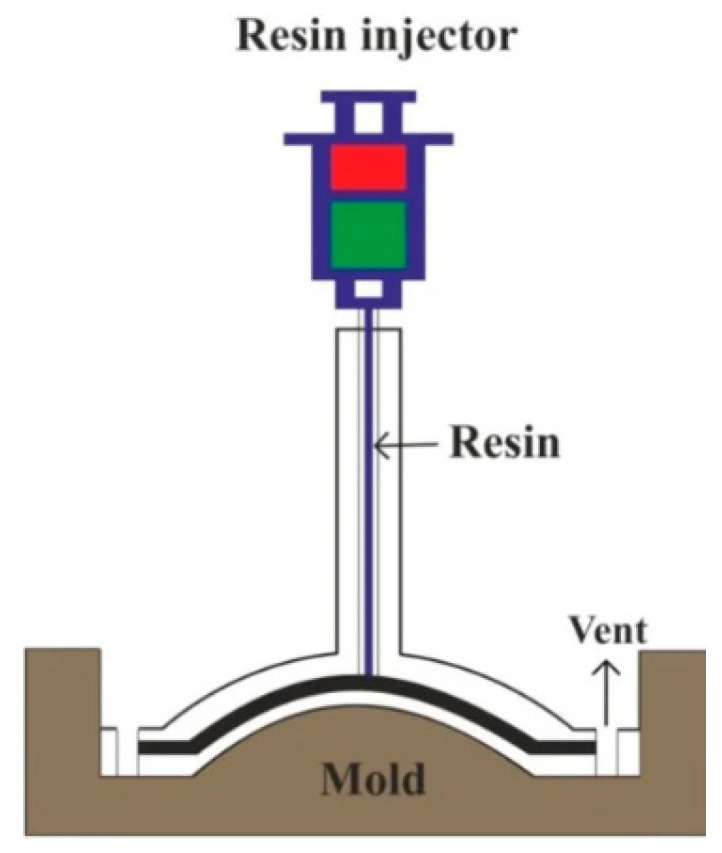
Schematic representation of resin transfer. Reprinted with permission [38].

This technique is a closed mold process in which the lower mold cavity is filled with fibers and then the mixture of resin, catalysts, and additives are injected under ambient temperature and high pressure. This leads to the wetting of the fibers. After that, the closure of the upper mold clamps the mold. Finally, the removal of the composite part allows the resin to be cured. This technique is based on preheating and loading polymer into the holding chamber instead of pouring it into the open mold [96]. The advantage of this technique is the effective use of fibers and matrix, and the suitability for viscous thermoset resin. Reinforcement in this technique can be found in the form of woven mats or strand mats. The air bubbles are usually prevented in this technique by the utilization of a vacuum which assists in the drawing of resin inside the cavity [97]. The resin transfer method is schematically depicted in Figure 7.

Although composite materials can be fabricated through a number of available manufacturing techniques, it is vital to find the most suitable technique for the fabrication of a specific composite material. Furthermore, the long-run targets encourage the development of advanced techniques to produce composite materials in high volume with lower cost and better performance.

## 5. Crashworthiness of GFRP Composite Structures

Structural crashworthiness is a vital requirement in designing automotive parts [98]. Additionally, the energy-absorbing capacity plays a key role in the automotive industry as it can increase passenger safety. Crashworthiness refers to the vehicle’s response during impact. The less damage to the vehicle and the passengers indicates proper crashworthiness performance after the crash [99]. The crashworthiness is determined by crashworthiness indicators such as energy absorption (*E_a_*), and specific energy absorption (*SEA*). The *SEA* indicates the energy absorption per unit mass of the absorber:

(1) $$SEA=\frac{E_{a}}{m}$$

in which *E_a_* and *m* represent the energy absorption during the crash and the total mass of the structure, respectively. The *E_a_* defines the energy absorption during a crash:

(2) $$E_{a}=\int_{0}^{s}F(x)dx$$

in which *S* and *F* are defined as crash displacement and impact force, respectively. The higher *SEA* denotes the better energy-absorbing capacity. The composite materials convert the kinetic energy to deformation-adsorbed energy. The crashworthiness performance of composite-based materials is mainly dependent on the material composition, and manufacturing process, while the metallic energy adsorbers convert the kinetic energy (impact) to plastic deformation energy during a vehicle collision. Therefore, the proper combination of materials together with the manufacturing process must be properly selected. The GFRP composite has gradually found applications as impact-absorbent automotive sections mainly due to its low cost of material, and optimal impact performance [100,101].

The performance of the GFRP composite structure is significantly affected by the volume content of the fibers and the staking sequence. In this regard, Solaimurugan et al. [102] have found that the increase in axial fiber content (below 68%) during axial impact increased the SEA of GFRP composite and the SEA was decreased beyond that content. Kathiresan et al. [103] have reported that decreasing the semi-apical angle in the GFRP conical tube increased the SEA. In another study, Hu et al. [104] studied the influences of fiber orientation on the crushing performance [105] of GFRP composite and their findings revealed that the fiber orientation did not show any significant effect on the SEA of the circular tube. The shape of GFRP can also influence the SEA. In this regard, Zhang et al. [106] investigated the SEA of different configurations and showed the highest SEA for hollow circular GFRP tubes compared to conical and square tubes.

Hybridization was also used to fabricate composite structures for crashworthy applications. In this regard, Ghafari-Namini et al., have stitched GFRP and CFRP composites and studied the effect of hybridization on crashworthiness [107]. Their results revealed that the energy absorption of the composite was improved over the unstitched one through the increase in the stitching of the composite box. In another report, Bakar et al., fabricated the hybrid composite by combining kenaf-glass/epoxy composite and showed a slightly lower SEA than that of the GFRP composite [108].

The composite sandwich structures can also be designed to fabricate energy-absorbing structures [109,110,111]. In this regard, Tarcholan et al. [112] fabricated a nested composite sandwich structure using woven fabric glass and carbon fibers together with expanded polystyrene (EPS) and epoxy resin. They have shown that a SEA of 47.1 kJ/kg was higher than that of 12.5–13.8 kJ/kg [104] for steel and 22–43 kJ/kg for aluminum [113]. Additionally, the internal thickness of nested tubes was found to play a key role in the energy-absorbing capacity of the composite. Tarcholan et al. [114] also fabricated composite sandwich structures using glass fiber, polystyrene foam, and epoxy resin. Their results showed a SEA of 32.6 kJ/kg which was higher than that of 10.3 kJ/kg for an empty composite tube with a tubular insert. Esnaola et al. [115] fabricated E-glass fiber-reinforced polyester and found a significantly higher SEA (38 kJ/kg) compared to 15–20 kJ/kg and 5–8 kJ/kg for aluminum and steel crash boxes, respectively [116].

In another report, Malcom et al. [66] stitched the E-glass core to S-glass fiber using kevlar and fabricated corrugated sandwich composite structures. Their findings showed the energy absorbed per unit volume in the range of 1.1–13 MJ/m^3^ which was higher than the empty core and sum of the foam. Chatterjee et al. [117] fabricated a sandwich composite structure using an E-glass 3D mat as the core and the layers of Kevlar with epoxy resin as the binder. They reported absorbed energy of 142.98 J for the sandwich composite filled with silica-based shear thickening fluid (STF), which was higher than that of 103.51 J and 85.40 J for the sandwich composite filled with PEG and blank 3D mat sandwich, respectively. This was because STF acted as a solid at the shear rate. Silva et al. [118] fabricated a three-phase composite by adding glass spheres and silica nanoparticles as the reinforcing phases into the polyamide 6/glass fiber and polypropylene/glass fiber. Their result showed that polyamide 6/glass fiber/glass sphere possessed a SEA of 51.7 kJ/kg which was higher than that of 26.3 kJ/kg for polypropylene/glass fiber/glass sphere.

## 6. Polymer Composite for Automotive Bumper Beam

### 6.1. Bumper System

**Figure 8:**
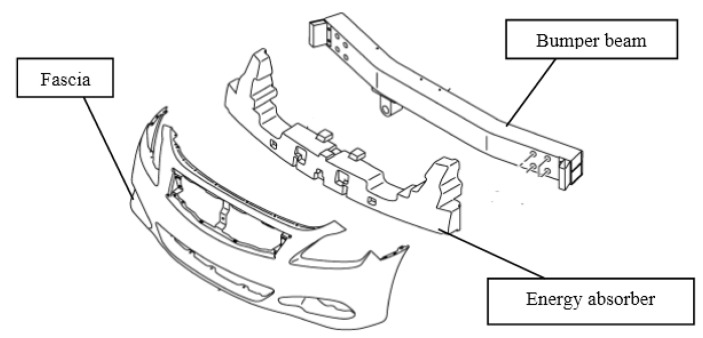
A schematic of the bumper system. Reprinted with permission [99].

The exterior trims are referred to as plastic constituents out of the car cabin. They should withstand impact loading [30,119]. In addition, the exterior body parts must possess high strength and stiffness to resist impact loadings during the collision which provides safety to the passengers. The bumper beam plays an important role in bearing sudden impact when a head-on collision occurs. Thus, two scenarios of low-impact and crashworthiness must be overwhelmed by bumper beams [120]. The bumper beam system is defined as the front and rear structure that absorbs energy during minor impact [121,122]. In most car crashes, the first part of the vehicle which goes under collision is the bumper system which may protect the body of the car and the passengers. It should not only be sufficiently deformable to absorb impact energy but also rigidity and strength to protect the vehicle parts and reduce the risk of injury. The forward bumper system should be stronger than that of the backward one to provide safety for drivers. Figure 8 shows the main parts of the bumper system including the fascia, energy absorber, bumper beam, and rails, as well as the cooling system support [123]. The non-structural aesthetic constituent in this system is fascia which cannot tolerate impact energy and reduces the aerodynamic drag force while the energy-dissipating part is the energy absorber which absorbs the kinetic energy during a collision. Weight, manufacturability, and reparability are among the key factors in selecting the bumper system [124].

By looking at the constituents of the bumper subsystem, the fascia or bumper cover, energy absorber or bumper foam, and reinforcing beam or bumper beam are related to the occupants and pedestrians. The fascia or bumper cover is designed for efficient aerodynamic performance and is lightweight. This is usually made up of polyurethane, polypropylene, and polycarbonate. The kinetic energy from vehicle collision is also adsorbed by the bumper foam. Finally, the key constituent in the bumper subsystem is the bumper beam which adsorbs the kinetic energy and protects the vehicle. It absorbs low and high-impact energy via bending resistance and collision, respectively [125]. The impact collision energy is absorbed in a controlled manner through the bumper beam before energy is transferred to the occupants. Deflection and intrusion are the two key parameters in the bumper beam. The former is determined as the maximum internal deformation of the bumper beam subsequent to the crash event. It is assumed that low deflection is required for the bumper beam system for passenger safety as there should be no contact between the deformed beam and the other parts of the automobile after the crash [126]. On the other hand, the latter is defined as the relative distance between the bumper beam part with the impact barrier section during the accident. Less intrusion is favored as the risk of injury for the passenger by the automotive hit is reduced [127].

### 6.2. Material Selection for Bumper Beam

The proper selection of material plays an important role in the development of bumper beams. The improper selection of material causes poor performance and failure which requires frequent maintenance. This further leads to an increase in cost. To select the proper material for the bumper beam, the type (e.g., axial, bending) and mode of loading (e.g., static, dynamic), operating conditions (e.g., temperature), manufacturing process as well as cost need to be considered [128]. Additionally, economic issues, environmental limitations, and mechanical and chemical properties affect material selection for bumper beams [129,130]. Mallick [128] and Edwards [130] have reported the vital criteria that need to be considered for the selection of suitable material for the bumper beam. Nonetheless, the nature of the criteria could make them incommensurable. Thus, a systematic approach is vital to obtain the optimal material. Therefore, the selection of optimal materials from various lists of materials is one of the most difficult tasks that design engineers are facing. This task has been made easier for design engineers by the development of multi-criteria Decision Making (MCDM) tools. Some of the leading MCDM tools used in the selection of automotive materials is comprised of Similarity of Ideal Solutions (TOPSIS) [131], Analytical Hierarchy Process (AHP) [132], Knowledge Base System (KBS) [133], and Fuzzy Multi-criteria Analysis [134], and TOmada de Decisao Interativa Multicriterio (TODIM) [135]. In this regard, Sapuan et al. [123] employed a weighted objective method to select the material for the bumper beam while Hosseinzadeh et al. [136] selected material for the bumper beam based on cost and impact loading using LS-DYNA ANSYS. Zeng et al. [137] applied Fruit Fly Optimization Algorithm (FOA) on a composite bumper beam and found that the composite bumper beam is 4.84% lighter than the steel counterpart with a 3.6 times larger peak value of energy absorption. Zindani et al. [120] have applied TODIM on various GFRP composites and found that glass fiber-reinforced epoxy is the optimal composite material for bumper beam application. Hamabli et al. [132] also applied AHP on different polymer composite materials and found that glass fiber-reinforced epoxy is the most appropriate material for bumper beam application. Osokoya et al. [26] chose different materials, using CES EduPack 2015, and found that glass fiber-reinforced polypropylene was more competitive in terms of cost/kg and has as many high mechanical properties as steel and aluminum. The estimated cost for glass fiber-reinforced polypropylene was found to be 2.03–2.68 (€/kg) which was the nearest to aluminum alloy with 1.32–1.46 (€/kg). This indicated that GFRP composite is the nearest alternative material when the cost is taken into consideration.

### 6.3. Computer-Aided Analysis

The automotive industry is depending on finite element analysis (FEA) in the development of products [138]. Physical tests are costly and time-consuming. However, the design can be analyzed in detail using FEA and this saves time and money through the reduction in the number of required prototypes. In addition, a high number of simulations can be performed to obtain satisfactory results before conducting the real physical test.

The car deflection can be approximately determined by FEA by analyzing energy absorption during impact. The analysis of energy absorption in real impact loading relies on different parameters which makes it very complicated. The FEA can be used to investigate the effects of design parameters on the weight, cost, and functional properties of new car models. The simulation of FEA is currently carried out by using different software including ANSYS, LS DYNA, ABAQUS, etc. In recent years, the glass mat thermoplastic (GMT) made of a unidirectional or woven GFRP composite laminate has been used in the manufacturing of commercial bumper beams due to the excellent energy-absorbing capability of impact energy as well as the lower weight compared to metallic counterparts [139]. Hosseinzadeh et al. [136] studied the impact behavior of commercial GMT bumper beams using ANSYS LS-DYNA under low-velocity impact. Their findings showed very good impact behavior compared to conventional materials including aluminum and steel. The conventional materials failed and revealed manufacturing difficulties which were ascribed to the strengthening ribs. In another study, Cheon et al. [140] investigated the mechanical properties of glass fiber epoxy composite by using ANSYS and their results showed a 30% reduction in weight compared to a steel bumper beam. Marzbanrad et al. [141] fabricated a front bumper beam from GMT and SMC and analyzed the impact behavior using LS-DYNA. They have shown that SMC was suggested to replace GMT due to its lower cost, easier production, high strength, and rib removal. In contrast, GMT showed manufacturing difficulties due to rib strengthening. Belingardi et al. [142] compared the energy-absorbing capability of E-Glass/epoxy pultruded bumper beams with steel using ABAQUS. Their findings revealed that a pultruded bumper beam possessed a comparable energy-absorbing capability to steel while showing better progressive failure with reduced peak load indicating its role as a safety component. A summary of the FEA modeling of composite as the automotive bumper beam is summarized in Table 6.

### 6.4. Glass Fiber Reinforced Polymer Composite Bumper Beam

Previously, steel, aluminum, and plastics have been commonly used as car bumper beams [7,8,26]. Steel is an appropriate material for bumper beam application due to its strength, stiffness, and high energy-absorbing capability. In addition, its mass production is very feasible. The aluminum grades ranging from mild strength to high strength steel as well as ultra-high strength steel (UHSS) can be used by the manufacturers to make a bumper beam. Aluminum is another common material for car bumper beams. It can save weight by up 50% when compared to steel, while retaining its performance, making it cost-effective [143]. Additionally, it can be mass-produced and meets the requirement for energy absorption [144].

Nonetheless, polymer composite material has replaced the above-mentioned traditional materials. GFRP composite has been used by car manufactures as it can provide lower weight, and lower energy consumption, as well as a higher energy-absorbing capability [145]. The use of GFRP composite has been extensively used for impact-induced automotive components due to its excellent impact performance and low-cost. Nevertheless, its major drawback for automotive applications is un-recyclability [146,147]. In the following section, the role of glass fiber as reinforcement in the composite bumper beam is discussed. In a study, Cheon et al. [140] used the glass fiber fabric-epoxy composite as the bumper beam with an elbow section made up of carbon-fiber epoxy. Their results showed a weight reduction of 30% for composite bumper beams compared to steel bumper beams and the bending strength was not compromised. In another study, Clark et al. [148] have shown that the inclusion of 40% glass led to a great enhancement in the stiffness of the composite with a fiber orientation of 45° compared to a random orientation. In another work, Prabhakaran et al. [149] designed a novel GFRP composite using E–glass/epoxy bidirectional laminate through hand lay-up and this resulted in a weight reduction of 53.8% compared to a steel bumper without comprising the strength. Shakirudeen et al. [150] fabricated GFRP composite using E-glass bidirectional laminate and epoxy resin by the hand lay-up process. Their findings showed that GFRP composite with 40% glass possessed an impact resistance of 100 kJ/m^2^ and a weight reduction of 60% compared to the steel bumper. In another study, Dakina et al. [151] reinforced polypropylene by the inclusion of various percentages of glass (0 to 70%) fibers and used it as a car bumper. It was found that the impact resistance was enhanced from 85 kJ/m^2^ to 498 kJ/m^2^. Witayakran et al. [152] have used the glass fiber content of 0 to 10% weight in epoxy resin and have shown a higher impact resistance for GFRP composite compared to that reinforced with oil palm fruit bunch (EFB). The higher impact resistance of GFRP composite was ascribed to the higher aspect ratio of glass fiber compared to EFB. Virgillito et al. [86] used different optical analyses, IR-thermography, and tomographic to assess the material damage of the GFRP composite and E–glass-reinforced epoxy matrix (laminated plate). Their findings showed that the impact velocity changed the way that materials absorbed energy. This was mainly attributed to the strain rate of the material. The increase in impact velocity from 1.5 m/s to 6 m/s led to an increase in SEA from 2.8 kJ/kg 3 kJ/kg using thermographic and tomographic methods. However, the optical method did not show any significant SEA change. El Haji et al. [153] fabricated the GFRP composite using post-consumer polypropylene car bumper waste (PP–CBW) and short glass fiber using melt processing and enhanced the interfacial bonding by coupling agents such as Retain and maleic anhydride-grafted linear low-density polyethylene (LLDPE–g–MA). They have shown that the Retain resulted in a higher impact property than LLDPE–g–MA. The incorporation of 20% glass fiber in the presence of Retain led to an increase in impact resistance from 7 kJ/m^2^ to 14.4 kJ/m^2^. In another study, Du et al. [154] fabricated 40% long glass fiber-reinforced polypropylene by injection molding. They have shown that the composite bumper beam possessed a higher energy absorption ratio, lighter weight, and low cost compared to aluminum alloy and steel bumper beams. Duan et al. [155] fabricated long glass fiber-reinforced polypropylene (LGFRP) and showed that the LGFRP bumper beam possesses a higher SEA of 195 J/kg than the 81.34 J/kg for the steel bumper beam. Additionally, LGFRP revealed a weight reduction of 51–58% compared to traditional counterparts. In another study, Zeng et al. [137] wound glass fiber-reinforced epoxy resin on ultra-high strength steel (UHSS) and their findings showed a 4.84% weight reduction and a 1.36 larger energy absorption compared to that of the steel counterpart. A comparison between the pure glass fiber-reinforced polymer composite and conventional metallic counterpart is tabulated in Table 7.

The manufacturing process needs to be considered during the selection of material for the development of the bumper beam. The GMT and LFT have been used as commercial bumper beams in the automotive industry. Compression molding is the common manufacturing process that is used for the fabrication of GMT and LFT [163,164,165]. It should be mentioned that the shape of the bumper beam is one of the factors that need consideration in an automotive application. In this regard, the pultrusion process can be used to fabricate composite bumper beams with complex geometrical shapes [26,115,122,142]. Table 8 was added to the manuscript and compares the bumper beam made from pure glass fiber-reinforced polymer composite with that of its conventional metallic counterpart.

Hybridization means the combination of more than one type of fiber in a polymeric matrix. The hybrid composite can reduce the weak characteristic of both natural and synthetic fibers and provide greater stiffness and strengthen great impact energy absorption. The integration of a kind of reinforcement material with a mixture of two distinct matrices [166], a mixture of several reinforcing phases in a single matrix [167], or the combination thereof is utilized to fabricate the hybrid composites. The mechanical properties of hybrid composites are dependent on the properties of the fiber, fiber-matrix interfacial bonding, arrangement, and orientation of fibers [168]. The hybrid composite materials can be divided into natural-synthetic, synthetic-synthetic, and natural-natural polymer-based composites. The natural-synthetic hybrid composite balances mechanical strength and environmental sustainability. Additionally, the presence of fibers of various diameters in the hybrid composite leads to an effective stress transfer which is ascribed to the increase in the aspect ratio and the interfacial area between the matrix and the fiber [169]. The applications of natural fiber-reinforced composite in exterior sections of the vehicle are restricted due to their high hydrophilicity [41].

The hybridization can be divided into three groups: (1) inter-ply in which the improvement is made at the laminate level through stacking layers of different components, (2) intra-ply in which different parallel bundles are combined inside the piles, and (3) super in which the layers of polymer composite is stacked in a particular stacking order [170]. Pegoretti et al. [171] fabricated intra-ply hybrid composite using E-glass polyvinyl alcohol/polyester laminates and found a superior impact performance for intra-ply hybrid composite than inter-ply counterpart under low-velocity impact [172]. Hung et al. [173] mixed E-glass fiber carbon plain weave fiber with epoxy. Their results showed that the carbon/glass-fiber reinforced polymer composite fabricated by vacuum bagging minimized the risk of damage with carbon fiber on the surface while severe damage was observed on the hybrid composite with glass fiber on the surface.

The rationale behind using hybrid composite as a car bumper beam is to maintain the merits of fibers and simultaneously lessen their restrictions [174,175]. The combination of high-strength synthetic fibers such as glass fibers and natural fibers could be used to fabricate the hybrid composite. The limitations of natural fibers are compensated by the presence of synthetic fiber and in turn, the mechanical properties of polymer composite are enhanced [176]. In this regard, Kim et al. [139] designed a hybrid composite comprised of woven E-glass and carbon fibers in a polypropylene matrix, and their findings revealed an improved impact performance (reduce intrusion and deflection) and a 33% reduction in weight compared to that of conventional GMT. In another study, Route et al. [177] designed a hybrid composite car bumper comprised of a coir fiber mat and a 7% glass fiber mat in a polyester resin matrix with and without NaOH surface treatment. Their findings revealed that the incorporation of NaOH surface-treated fiber led to an increase in impact resistance from 576.0 J/m to 687.8 J/m. This increase in impact resistance was attributed to the toughness of coir fibers. Khalil et al. [178] fabricated the EFB/glass hybrid-reinforced polyester composite and found that 35% fiber content increased the impact resistance. Nonetheless, further increase in fiber content decreased the impact resistance which was ascribed to the inter-fiber interaction. In a study, Olorunnishola et al. [179] fabricated the hybrid composite by using the hand lay-up method comprised of natural jute and 10% synthetic glass fiber in a polypropylene matrix. The results showed an impact resistance of 12.6 J which was significantly higher than that of 9.6 J for commercial long glass fiber filled (GF–C). The combination of glass fibers with hemp fibers in epoxy resin have also shown desirable impact resistance for bumper beam application [180]. In another report, Davoodi et al. [181] fabricated the hybrid Kenaf/glass fiber-reinforced epoxy composite by modified sheet molding compound (SMC) technique for bumper beam application. They discovered that the impact resistance of the hybrid composite was 26 J/m which was almost half of the reported value for GMT. In another study, Davoodi et al. [182] fabricated Kenaf/glass fiber-reinforced epoxy composite by SMC technique and attempted to improve the impact resistance of the composite by using CBT thermoplastic toughening. Their findings showed that the impact was considerably improved and reached 40.2 J/m but it was still lower than that of GMT. Davoodi et al. [165] also fabricated Kenaf/glass fiber-reinforced epoxy composite by SMC technique and aimed to improve the impact resistance level of the composite by polybutylene terephthalate (PBT) toughening for bumper beam application. Their results revealed that the impact resistance of the PBT-toughened composite was enhanced from 26 J/m to 40.2 J/m compared to Kenaf/glass fiber-reinforced epoxy composite. However, it was still lower than that of GMT with an impact resistance of 50 J/m. This indicated that the impact properties of the composite need to be enhanced by the optimization of structural design parameters such as beam curvature and strengthening ribs. Mishra et al. [183] fabricated pineapple leaf (PALF)/glass fiber-reinforced polyester and sisal/glass fiber-reinforced polyester hybrid composite. They have shown that increasing the weight percentage of glass fiber from 0 to 8.6 increased the impact resistance from 68.23 J/m to 128 J/m indicating an 87% increase in impact resistance. Raghav Arvind et al. [184] fabricated GFRP composites comprised of glass fibers and epoxy resin by using the hand lay-up technique. It was revealed that the GFRP composites possessed an impact resistance of 14. J. David et al. [185] developed a hybrid composite of coconut fiber/glass fiber and a matrix of reinforced low-density polyethylene (RLDPE) using a compression molding technique for bumper beam application. They have shown that the impact resistance of coconut fiber/RLDPE was increased from 3.6 J to 4.8 J by increasing the glass fiber content. This was attributed to the hybridization and the presence of glass fibers well-bonded with RLDPE. Xue et al. [161] developed a long fiber-reinforced polypropylene (LGRF-PP) composite for bumper beam application using the hot-melt impregnation technique. Their findings showed a cost reduction, and mass reduction of 69% and 17.4%, respectively. Additionally, the composite showed a 6% increase in SEA compared to Al6061. In another study, Paramasivam et al. [186] fabricated glass/basalt reinforced composite using hand-lay-up and epoxy resin for lightweight automobile applications. They showed that the impact resistance was increased from 9.8% to 12.12%, which was attributed to the curing time, blend proportion and pressure.

Nachippan et al. [187] fabricated the hybrid GFRP composite using the hand lay-up technique. Their findings showed the composite made up of treated/untreated hemp/S-glass reinforced epoxy composite absorbed the significant impact and the deformation factor was extremely low under loading. Sreerama et al. [36] fabricated the GFRP composite using the hand lay-up technique and compared the impact performance with structural steel using the design and optimization process in sedan cars. Their findings have shown a 36% reduction in weight and 14 times greater deformation compared to structural steel. Joo et al. [156] fabricated the glass fiber-reinforced thermoplastic composite rod (3D–Tow)/over-molded long chopped glass fiber-reinforced thermoplastic (LFT) composite using E-glass and polypropylene. They have reported the insertion of 3D-Tow in the bumper assembly component led to an increase in energy absorption from 41.2 to 96.5 J indicating a 134% increase in energy absorption. In addition, the impact performance of the bumper was found to be proportional to the bond strength by finite element analysis (FEA) simulation.

Jeyanthi et al. [7] fabricated twisted kenaf/glass fiber-reinforced plastic (TFKLRT) polypropylene composite by injection molding technique for bumper beam application. They have shown that the impact resistance of the hybrid composite was found to be around 140 J/m which was considerably higher than that of 120 J/m for commercial long fiber thermoplastics (LFT). Atiqah et al. [188] fabricated the Kenaf/glass fiber-reinforced polyester resin composite by sheet molding compound technique. Their findings have shown that the surface-treated kenaf fibers by a mercerization process led to an impact resistance value of 146.43 J/m which was 11% higher than the composite made by untreated kenaf fibers. This was attributed to the mercerization process which enhanced the adhesion between the surface of the fibers and the matrix. Kwon et al. [189] fabricated polypropylene/continuous glass fiber (GCF) by a lamination process. Their results showed a high impact resistance of 500 J/m and the impact resistance was significantly affected by the polypropene content. However, the impact resistance of the composite in line with the machine direction was slightly higher than that of the transverse direction. In another study, Bakkal et al. [190] fabricated short glass fiber laminar and knitted glass fiber laminar composite materials for bumper beam applications. They have shown that the hybridization of low-density polyethylene (LDPE)/cotton with short and knitted glass fiber led to an impact resistance of 479 kJ/m^2^ and 464 kJ/m^2^ which was higher than that of 236 kJ/m^2^ for the commercial light truck (bumper) indicating a 200% increase in impact resistance. In another study, Haydar et al. [37] fabricated unsaturated polyester resin (UPE)/zirconium oxide (ZrO_2_)/E–glass and their results showed a significant increase in impact resistance. The addition of 2.5 wt.% ZrO_2_ showed an impact resistance of 73.1 kJ//m^2^ which was found to be higher than that of 49.7 kJ/m^2^ for commercial Chery bumper. Nonetheless, a further increase in the content of ZrO_2_ decreased the impact resistance due to the agglomeration of nanoparticles. Vijay Ramnath et al. [191] fabricated three different hybrid composites using GFRP composite and abaca, GFRP composite, and jute and their combination thereof. Their findings showed that GFRP/jute/abaca composite possessed an impact resistance of 12 J which was lower than that of 16 J and 15 J for GFRP/abaca GFRP/jute, respectively. A summary of hybrid GFRP for bumper beam application is shown in Table 8.

**Table 8:** A summary of hybrid GFRP composite for bumper beam applications.

Composite	Fabrication Technique	Major Findings	References
Kenaf/glass fiber-reinforced epoxy	Sheet molding	Lower impact resistance than GMT	[181]
CBT-toughened Kenaf/glass fiber-reinforced epoxy	Sheet molding	Significant increase in impact resistance	[182]
PBT-toughened Kenaf/glass fiber-reinforced epoxy	Sheet molding	Lower impact resistance compared to GMT	[192]
Twisted kenaf/glass fiber-reinforced plastic	Injection molding	Higher impact resistance than commercial LFT	[7]
Glass/carbon fiber-reinforced thermoplastics	–	33% weight reduction with improved impact performance	[139]
Jute/glass fiber-reinforced polypropylene	Hand lay-up	Superior impact resistance than commercial long glass fiber filled	[179]
Abaca/glass fiber-reinforced epoxy resin	Hand lay-up	Superior impact resistance compared to Jute/abaca glass fiber-reinforced epoxy resin	[191]

## 7. Impact Response of GFRP Composite Materials

The GFRP composite materials are vulnerable to impact damage compared to metals as the metals have ductility and intrinsic energy absorption [193]. Therefore, the energy-absorbing capacity and impact features of composite materials need to be improved to avoid structural failure [194]. The prediction of damage behavior of lightweight GFRP composite under impact load is important for the design and development of GFRP composite for the automotive component. This is because impact events can cause significant damage in composite structures with different failure modes comprising fiber breakage, matrix cracking, multi-delamination, and catastrophic failures [195,196]. Knowledge of damage mechanics for the load-bearing performance of composite structures can be found elsewhere [197,198]. The behavior of the GFRP composite under low and high velocities has been investigated and the findings showed that the more fibers, the more sustained impact energy [199,200]. The selection of materials for matrix and fibers affects the impact properties of fiber-reinforced composite subjected to impact loading. In this regard, the type of matrix material selected to fabricate composite material influences the load transfer between the fibers [201].

In general, four types of impact can be classified based on the impact velocity: (1) low-velocity (<11 m/s), (2) high-velocity (>11 m/s), (3) ballistic (>500 m/s), and (4) hyper-velocity (>2000 m/s) [202]. In low-velocity impact case analysis, the geometry of the target plays an important role in controlling the energy-absorbing capability of the composite. The composite material is also damaged under low-velocity impact but it can still function. On the other hand, the composite material is penetrated by the impactor under high-velocity impact. In addition, the energy dissipation due to failure occurs in a small zone which is ascribed to the more localized type of target response in high-velocity impact [203]. A large amount of impact energy can be absorbed and dissipated under impact loading in a wide range of damage and failure modes [204]. Most of the applied impact energy is absorbed when the composite structure behaves under an elastic regime. Several factors affect the elastic absorption of impact energy comprising: (1) matrix toughening, (2) fiber toughening, and (3) interface toughening [205]. The impact performance of composite materials could be affected by the type and properties of the fibers, fiber arrangement, stacking sequence, as well as volume fraction. Additionally, the geometry of the component and environmental conditions can also influence the impact response of composite materials [206]. In a study, Kim et al. [157] fabricated glass-fiber-reinforced polypropylene and showed that the impact response (intrusion and deflection) can be accurately predicted by using strain rate-dependent mechanical behavior.

The mechanism by which the damage is generated during impact needs to be understood to explore the key factors determining the structural performance of composite materials. The damage process of the composite is complex and this is ascribed to the uneven distribution of stress, as well as their anisotropic nature under the transient loading [207]. Additionally, the natural brittleness of composite materials causes energy absorption in the elastic state making them susceptible to impact failure [208]. The mechanics and mechanisms of composite failure under impact loading are divided into five main stages as follows: (1) cracking of matrix phase that leads to fiber/matrix interface debonding mode due to high shear stress, (2) transverse bending crack that is generally due to the high flexural stress in the bottom layers, (3) mesoscale interlaminar damage that appears as multiple delaminations due to the diversion of cracks in the interface area, (4) fiber breakage which is a failure damage mode under tension as well as fiber micro-buckling that normally occurs under compressive loads, and (5) penetration [209]. In stage 1, a rapid increase in load without noticeable damage is followed by matrix cracking [210]. In stage 2, the rapid spread of matrix cracking leads to interlaminar delamination (interfacial debonding) [211]. The factors that affect the impact resistance of composite materials are comprised of the matrix type, the thickness of the laminate, the lay-up sequence as well as gematrical conditions [212].

**Figure 9:**
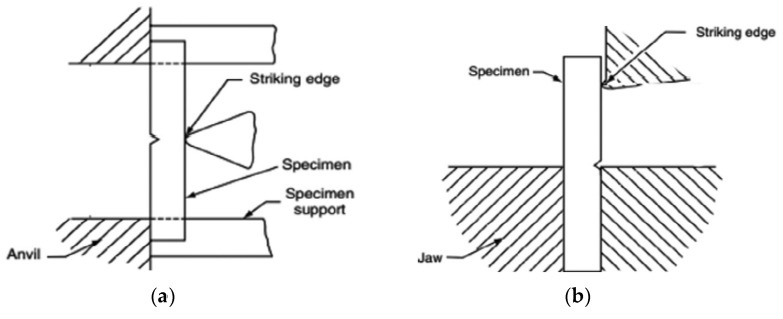
Schematic representation of (**a**) Charpy, and (**b**) Izod impact tests. Reprinted with permission [58].

Various mechanical tests under measured laboratory conditions elaborate on the performance of polymers and polymer composite material. Some of the commonly used tests are tensile, compressive, flexural, and impact [213]. The impact energy is defined as the amount of work performed to break the material. Upon impacting the test sample by the striker (impactor), the energy is absorbed by the sample until it is fractured [214]. Impact testing is carried out to determine the impact energy of a material. In other words, the total amount of energy that can be absorbed by the composite is measured by the impact test. The dynamic impact response of polymer composites is evaluated using available impact testing methods including high velocity, drop weight, Charpy, and Izod impact tests. The Charpy and Izod impact tests are conducted based on the ASTM D6110 and ASTM D256, respectively. The impact energy (resistance) of a material is expressed in J/m or optional unit kJ/m^2^ [215,216]. In the Charpy test, a horizontally supported beam and a vertical cantilever beam are used to conduct Charpy and Izod tests, respectively. The impact toughness and the Notch sensitivity are the properties that are derived from the impact test. The Charpy and Izod tests are schematically depicted in Figure 9.

A comparison between the impact resistance of commercial bumper beams and GFRP composite-based bumper beams revealed that sisal/glass fiber-reinforced polyester hybrid composite, NaOH–treated coir-polyester/glass fiber mat Acrylonitrile-grafted coir-polyester/glass fiber mat showed a significantly higher impact resistance compared to that of the commercial counterpart (using Izod test), while the impact resistance of GFRP composite-based bumper beams such as hybrid Kenaf/glass fiber-reinforced epoxy composite, CBT–toughened Kenaf/glass fiber-reinforced epoxy, and PBT-toughened Kenaf/glass fiber-reinforced epoxy was still lower than that of commercial GMT (Table 9).

## 8. Conclusions and Future Outlook

Over the past decades, growing demands for the enhancement of fuel efficiency and weight reduction have motivated vehicle manufacturers to pursue a weight-saving revolution. Accordingly, the replacement of conventional materials such as cast iron and steel with lightweight materials was found to be an effective strategy for weight reduction. This review article presented an overview of the energy-absorbing capability and impact resistance of synthetic and natural GFRP composite for potential car bumper beam application. From the literature review, weight reduction, improved energy-absorbing capacity, impact resistance, and study on progressive damage process and long-term damage history of the bumper structure, were identified for the GFRP bumper compared to commercial bumper and conventional materials.

Nonetheless, the commercial success of GFRP composite as a bumper beam requires large production and further acceptance by multisector automotive industries. For this purpose, analytical tools such as Similarity of Ideal Solutions (TOPSIS) [131], Analytical Hierarchy Process (AHP) [132], and Fuzzy Multi-criteria Analysis [134] should be implemented to assist engineers in a wise selection of matrix, and fibers for bumper application. This indicates that both academia and industry still need to make a significant endeavor to develop versatile lightweight GFRP composites and transfer them into practice with large-scale production.

## Figures and Tables

**Table 1:** Examples of GFRP constituents in automotive applications.

Application	Manufacturer	Highlights	Reference
Leaf spring	GM Corvette	80% weight reduction compared to steel counterpart	[32]
Leaf spring	Chevrolet Corvette C4	15 kg weight reduction	[33]
Suspension spring	Audi AG	40% weight reduction compared to steel counterpart	[34]
Instrument and indoor panel modules	Landover Evoque	–	[35]
Door module	Faurecia Jeep Liberty SU V	–	[35]
Fluid filter module	Daimler AGT–Mercedes	–	[35]
Bumper beam	Sedan	36% reduction in weight compared to structural steel	[36]
Bumper beam	–	significant increase in impact resistance	[37]

**Table 2:** A comparison between natural and synthetic fibers.

Properties	Synthetic Fiber	Natural Fiber	Reference
Impact	High	Poor	[57]
Cost	High	Low	[58]
Strength	High	Low	[50]
Recyclability	Poor	High	[41]

**Table 3:** A comparison between thermosetting and thermoplastic matrix.

Properties	Thermoset	Thermoplastic	Reference
Viscosity	Low	High	[58]
Impact strength	Low	High	[76]
Melting point	High	Low	[58]
Modulus	High	Low	[48]

**Table 4:** A summary of mechanical properties of the polymer matrix.

Matrix	Strength (MPa)	Reference
Polypropylene	26.41	[77]
Polyethylene	20–35	[78]
Nylon	500	[79]
Polyether ether ketone	100	[78]
Polyester	55–60	[78]
Polystyrene	25–69	[78]
Phenolic	35–60	[78]
Epoxy	50–100	[80]

**Table 5:** A summary of physical and mechanical properties of different glass fibers [90].

Fiber	Density (g/cm^3^)	Tensile Strength (GPa)
E–glass	2.58	3.44
C–glass	2.52	3.31
S–glass	2.46	4.89
A–glass	2.44	3.31
D–glass	2.11	2.41
R–glass	2.54	4.13

**Table 6:** A summary of FEA modeling of composite bumper beam obtained from the literature.

Software	Study	Findings	Reference
LS DYNA	Crashworthiness of SMC and GMT composite bumper beam	Th increase in the thickness of the bumper beam and the addition of ribs increased the rigidity and impact force of bumper	[141]
ABAQUS	Crashworthiness of frusta made up of glass fiber/epoxy laminated thin-walled composite	A close match between the experimental results and the FEA modeling	[103]
ABAQUS	E–Glass/epoxy pultruded bumper beam	Comparable energy-absorbing capability with steel while showing better progressive failure with reduced peak	[142]
ANSYS LS-DYNA	Analysis of the impact behavior of GMT, aluminum, and steel under low-velocity impact	Very good impact behavior compared to aluminum and steel	[136]
ANSYS	Mechanical properties of glass fiber epoxy composite bumper beam	30% reduction in weight compared to steel bumper beam	[140]

**Table 7:** A literature summary of the comparison between GFRP and the conventional metallic counterpart.

Composite	Manufacturing	Control	Major Findings	Reference
Glass fiber-reinforced epoxy	–	Steel	36% weight reduction and 14% increase in deformation	[36]
Long E-glass Fiber-reinforced polypropylene	Air-lay process	Steel	51–58% weight reduction and two times greater SEA	[155]
GMT	Compression molding	Steel, Aluminium	Good impact behavior reduction of material, ease of manufacturing	[136]
E-glass epoxy pultruded	Pultrusion	Steel, E-glass fabric	Comparable energy absorption with steel and E-Glass fabric, better progressive failure mode, and reduced peak load	[122,142]
Over-molded chopped glass fiber polypropylene/continuous glass fiber polypropylene composite	3D Tow-printing	–	Explicit predication of mechanical behavior by FEA	[156]
Glass fiber-reinforced polypropylene	Prepreg impregnation	–	Accurate prediction of impact response through strain rate-dependent mechanical properties	[157]
GMT	Compression molding	Steel	Little effect on the crashworthiness and 41 kg weight reduction	[158]
Glass fiber-reinforced polyamide	Injection molding	Steel	45% weight reduction and better recyclability	[159]
E–glass fiber-reinforced epoxy resin	Hand lay-up	Steel	64% increase in a safety factor and higher load-withstanding capability	[160]
E–glass fiber-reinforced polyester	UV–cured pultrusion	Steel, aluminum	Significantly higher SEA	[115]
Glass fiber-reinforced polypropylene	Pultrusion	Steel	Cost-competitive and higher specific strength	[26]
Long glass fiber-reinforced polyamide	Injection molding	Steel, aluminum	Higher SEA, lighter weight, and lower cost	[154]
Long glass fiber-reinforced polypropylene	Hot-melt impregnation	Aluminum	6% increase in SEA, 69% decrease in cost, and reduced peak force	[161]
E-glass reinforced-epoxy resin bidirectional laminate	Hand lay-up	Steel	60% weight reduction and higher impact resistance	[150]
E-glass reinforced-epoxy bidirectional laminate	Hand lay-up	Steel	53.8% weight reduction	[149]
Glass fiber fabric-reinforced epoxy	Prepreg impregnation	Steel	30% weight reduction	[140]
GMT	Compression molding	Chromium-coated mild steel, aluminum	Higher impact resistance and low deformation	[162]

**Table 9:** A summary of the impact resistance of GFRP composite for bumper beam applications.

Material	Impact Test	ASTM	Impact Resistance (J/m)	Control	Impact Resistance (J/m)	Reference
hybrid Kenaf/glass fiber-reinforced epoxy composite	Izod	D256–04	26	Commercial GMT	50	[181]
CBT-toughened Kenaf/glass fiber-reinforced epoxy	Izod	D256–04	40.2	Commercial GMT	50	[182]
PBT-toughened Kenaf/glass fiber-reinforced epoxy	Izod	D256–04	40.2	Commercial GMT and hybrid Kenaf/glass	50 26	[192]
Twisted kenaf/glass fiber-reinforced plastic	Izod	D256–04	140	Commercial LFT	120	[7]
Jute/glass fiber-reinforced polypropylene	Izod	D256–04	12.6	Commercial GF–C	9.6	[179]
NaOH-treated coir-polyester/glass fiber mat Acrylonitrile-grafted coir-polyester/glass fiber mat	Izod	D256–04	687.8 629.8	Untreated Untreated	576.0 576.0	[177]
PALF/glass fiber-reinforced polyester and sisal/glass fiber-reinforced polyester hybrid composite	Izod	D256–04	128 148.5	PALF/polyester Sisal/polyester	68.12 110.25	[183]
GFRP is comprised of glass fibers and epoxy resin and glass fibers and epoxy resin containing aluminum	Charpy	–	20	GFRP	14	[184]
RLDPE/coconut fiber/glass fiber	Charpy	578/D578M	4.8	RLDPE/coconut fiber	3.6	[185]
GFRP/abaca	Charpy	D256.0	16	GFRP/jute GFRP/jute/abaca	15 12	[191]

## Data Availability

Not applicable.
